# Incomplete resection and linitis plastica are factors for poor survival after extended multiorgan resection in gastric cancer patients

**DOI:** 10.1038/s41598-017-16078-x

**Published:** 2017-11-17

**Authors:** Hua Xiao, Min Ma, Yanping Xiao, Yongzhong Ouyang, Ming Tang, Kunyan Zhou, Yuan Hong, Bo Tang, Chaohui Zuo

**Affiliations:** 1 Department of Gastroduodenal and Pancreatic Surgery, Hunan Cancer Hospital and The Affiliated Cancer Hospital of Xiangya School of Medicine, Central South University, Changsha, 410013 China; 2 Department of Admissions and Employment, Changsha Health Vocational College, Changsha, 410010 China

## Abstract

The aim of this retrospective study was to analyze the morbidity, mortality, and survival rates of extended multiorgan resection (EMR) for locally advanced gastric cancer patients compared to gastrectomy alone and a palliative operation. 893 locally advanced gastric cancer patients without distant metastasis had surgery including gastrectomy alone (GA group, n = 798), EMR resection (EMR group, n = 75), and palliative operation (palliative gastrectomy or gastrojejunostomy (PO group, n = 20)). Postoperative mortality and complication rates in the EMR group were significantly higher than in the GA group (2.7% vs 0.4%, P = 0.010 and 25.3% vs 8.1%, P < 0.001, respectively), but similar in the PO group. The median survival time of the EMR group was significantly longer than in the PO group (27 months vs 11 months, P = 0.020), but significantly worse (P = 0.020) than in the GA group (44 months). Incompleteness of resection (R1) and linitis plastica were independent prognostic factors for survival in the EMR group. Three different gastric cancer surgeries led to different postoperative mortality and complication rates. EMR had a better survival rate compared with PO while GA had the longest survival time with the lowest mortality and complication rates.

## Introduction

Gastric cancer is one of the most common forms of cancer detected worldwide and is a leading cause of death. In general, it occurs most prevalently in Eastern Asia, particularly in Korea, Mongolia, Japan and China^1^. Unfortunately, gastric cancer typically presents at a locally advanced stage, even invading into adjacent organs in many of our patients in China. For these patients, extended multiorgan resection (EMR) is advocated as the operation of choice for achieving R0 resections, which has been identified as an important indicator of longer term survival in patients undergoing curative surgery for gastric cancer^2–4^ and the long-term outcomes of palliative resection or non-surgical treatments such as chemotherapy for locally advanced gastric cancer are dismal^5^. On the other hand, some studies concluded that EMR should be cautiously performed in strictly selected patients because of increased postoperative morbidity and mortality^6,7^. As far as we are aware, no studies have directly compared the outcomes of EMR and palliative operations for gastric cancer in which invasion of nearby organs was suspected. When designing treatment for patients with advanced gastric cancer the optimal surgical procedures remain unclear. In the present study, we retrospectively assessed the effectiveness of EMR compared with gastrectomy alone or palliative surgery in patients with local gastric cancer.

## Patients and Methods

From November 2010 to September 2015, 893 patients diagnosed with locally advanced gastric cancer without distant metastasis underwent surgery in the Department of Gastroduodenal and Pancreatic Surgery, Affiliated Cancer Hospital of Xiangya Medical School, Central South University. The inclusion criteria were: (1) patients diagnosed with locally advanced gastric cancer who underwent gastrectomy with a curative intent or a palliative operation including gastrojejunostomy or palliative gastrectomy, (2) patients without distant metastasis, or other malignancies occurring at the same time, (3) patients who had comprehensive and complete clinical records, postoperative pathological and follow-up data. The diagnosis of the cancer stage was based on clinical data, including an enhanced computed tomography (CT) scan, endoscopic ultrasonography (US), and intraoperative assessments. Laparoscopy was performed in patients with linitis plastica in order to detect peritoneal metastasis. In patients with ascites, cytology of the intraperitoneal fluid was performed, while positive cytology was considered as M1 cancer and excluded from this study. EMR was suggested to all patients with locally advanced gastric cancer and suspected invasion into the adjacent organs to achieve R0 resection, but the final choice between EMR and PO has been made by the patients.

Our study was performed in accordance with the Declaration of Helsinki regarding the ethical principles for medical research involving human subjects. This study was approved by the Ethics Committee for Clinical Pharmacology in Central South University, and all patient data were kept strictly confidential. All participants signed informed consent forms.

### Surgical procedures

All of the surgical procedures were carried out by highly qualified surgeons. The standard surgical procedures for advanced gastric cancer with curative-intent included D2 or D2 + lymphadenectomy^8^. The definition of EMR employed was simultaneous en-bloc resection of the stomach and the suspected invading organs. Postoperative morbidity and mortality were graded using a modified Clavien–Dindo classification of surgical complications^9^. Postoperative mortality was defined as deaths that occurred within 30 days after surgery. The TNM stage was defined by the 7th TNM AJCC/UICC guidelines^10^. Standard treatment regimens for neoadjuvant chemotherapy or adjuvant therapy after surgery were fluorouracil (such as S-1) + platinum (such as oxaliplatin) chemotherapy for 6 months^11^.

### Statistical analysis

Statistical analyses were performed using IBM SPSS Statistics for Windows (Ver. 19. IBM Corp., Armonk, NY). Demographic data that included the clinicopathological characteristics of the patients, the perioperative outcome, and overall survival rates were retrospectively collected and analyzed. All continuous variables are expressed as the mean ± s.d. and potential differences between groups were assessed using an independent-samples *t*-test or a Mann–Whitney U-test. Categorical variables are reported as the total number of cases and prevalence, and differences between groups were compared by Fisher exact or χ^2^ tests. Survival curves were constructed from the observed survival times using the Kaplan-Meier procedure. A log-rank test was used to test for any significant differences in the survival rates. A Cox regression model was used for multivariate analysis. A *P*-value < 0.05 was considered to be statistically significant.

## Results

### Basic demographic information of enrolled patients

In total, 1,594 patients were operated on for gastric cancer from November 2010 to September 2015. Of these, 893 patients who underwent surgical treatments were enrolled in our study after being diagnosed with advanced local gastric cancer without the complication of distant metastasis. Patients were allocated into 3 groups according to the surgical approaches used: radical gastrectomy alone (GA group, *n* = 798), extended multiorgan resection (EMR group, *n* = 75) and a palliative operation group including palliative gastrectomy and gastrojejunostomy (PO group, *n* = 20). The mean patient age was 54.6 ± 10.7 years (range, 19–83 years, 893 patients); 608 were men (68.1%) and 285 were women (31.9%). In the PO group, 11 patients (55.0%) with bleeding or pyloric obstruction underwent palliative gastrectomy and the remaining 9 patients with pyloric obstruction underwent gastrojejunostomy.

The proportion of male, American Society of Anesthesiologists score ≥ 3, any comorbidity, neoadjuvant chemotherapy, differentiation type, mean age and body mass index (BMI) were comparable among the 3 groups **(**Table 1 **)**. Preoperative albumin and hemoglobin levels were both significantly increased in the GA group patients compared with the EMR and PO groups. Upper third located cancer and linitis plastica were more common in the EMR group (38.7%) and as a result, a significantly greater number of total gastrectomy operations were performed (*P* < 0.001). The numbers of harvested lymph nodes were significantly greater in the EMR group when compared with the GA group. The majority of the EMR group presented with a higher T and TNM stage, although the N stage distribution was not significantly different from that found in the GA and EMR groups. Prolonged operative time and post-operative hospital stays, increased intraoperative blood loss, and perioperative blood transfusion were mainly observed in the EMR group.

**Table 1:** Clinicopathological characteristics of the entire study cohort stratified by extent of resection.

	GA group (*n* = 798, 89.4%)	EMR group (*n* = 75, 8.4%)	PO group (*n* = 20, 2.2%)	χ^2^ or *t* value	*P*-value
Preoperative characteristics					
Sex (Male)	534 (66.9%)	58 (77.3%)	16 (80.0%)	4.76	0.090
Age (years)	54.3 ± 10.9	56.6 ± 11.3	55.6 ± 10.7	1.57	0.210
BMI (kg/m^2^)	21.5 ± 2.9	20.9 ± 3.1	21.0 ± 2.5	1.40	0.250
ASA ≥ 3	122 (15.3%)	16 (21.3%)	2 (10.0%)	2.39	0.300
Any comorbidity	230 (28.8%)	30 (40.0%)	4 (20.0%)	5.01	0.080
Neoadjuvant chemotherapy	36 (4.5%)	5 (6.7%)	2 (10.0%)	1.90	0.390
Adjuvant chemotherapy	(63.2%)	(69.3%)	(70%)		0.180
Preoperative albumin (g/L)	36.8 ± 4.8	35.3 ± 5.0	34.1 ± 8.0	5.69	0.004
Preoperative hemoglobin (g/L)	117.0 ± 24.2	106.3 ± 26.5	99.1 ± 31.0	11.20	<0.001
Complication due to the tumor	232 (29.7%)	25 (33.3%)	20 (100.0%)	40.08	<0.001
Pyloric obstruction	150 (18.8%)	13 (17.3%)	15 (75.0%)	38.96	<0.001
Bleeding	82 (10.3%)	12 (16.0%)	5 (25.0%)	6.30	0.040
Surgery					
Resection type				426.34	<0.001
Proximal subtotal	34 (4.3%)	7 (9.3%)	1 (5.0%)		
Distal subtotal	552 (69.2%)	29 (38.7%)	6 (30.0%)		
Total gastrectomy	212 (26.6%)	39 (52.0%)	4 (20.0%)		
Gastrojejunostomy			9 (45.0%)		
Intraoperative blood loss (mL)	201.6 ± 106.6	353.9 ± 310.7	155.5 ± 101.9	45.00	<0.001
Operation time (min)	203.0 ± 50.6	257.7 ± 76.3	191.0 ± 58.4	36.98	<0.001
Pathology					
Tumor location				33.94	<0.001
Upper third	74 (9.3%)	18 (24.0%)	2 (10.0%)		
Middle third	171 (21.4%)	20 (26.7%)	6 (30.0%)		
Lower third	507 (63.5%)	26 (34.7%)	10 (50.0%)		
Linitis plastica	46 (5.8%)	11 (14.7%)	2 (10.0%)		
Tumor diameter (cm)	4.5 ± 2.3	5.4 ± 2.8	—	3.19	0.070
T stage				8.90	0.003
T3	152 (19.2%)	4 (5.3%)	—		
T4	645 (80.8%)	71 (94.7%)	—		
N stage				0.65	0.880
N0	186 (23.3%)	16 (21.3%)	—		
N1	142 (17.8%)	15 (20.0%)	—		
N2	200 (25.1%)	16 (21.3%)	—		
N3	270 (33.8%)	28 (37.3%)	—		
Lymph node harvested	19.9 ± 8.5	23.0 ± 9.0	—	3.02	0.003
Differentiation type				1.49	0.480
Poor-undifferentiated	684 (85.7%)	61 (81.3%)	16 (80.0%)		
Well-moderate differentiated	114 (14.3%)	14 (18.7%)	4 (20.0%)		
TNM stage				11.36	0.001
II	188 (23.6%)	5 (6.7%)	—		
III	610 (76.4%)	70 (93.3%)	—		
Surgical margin				27.10	<0.001
Negative (R0 resection)	780 (97.74%)	65 (86.67%)			
Positive (R1 resection)	18 (2.26%)	10 (13.33%)			
Postoperative outcomes					
30-day mortality	3 (0.4%)	2 (2.7%)	1 (5.0%)	11.13	0.004
Complications	65 (8.1%)	19 (25.3%)	2 (10.0%)	23.28	<0.001
Clavien complications ≥ IIIa	25 (3.1%)	10 (13.3%)	1 (5.0%)	18.49	<0.001
Perioperative blood transfusion	181 (22.7%)	44 (58.7%)	8 (40.0%)	48.09	<0.001
Post-operative hospital stays (days)	11.9 ± 4.5	14.8 ± 10.7	10.2 ± 2.9	11.75	<0.001

GA, gastrectomy alone; EMR, extended multiorgan resection; PO, palliative operation; BMI, body mass index; ASA, American Society of Anesthesiologists.

Preoperative albumin and hemoglobin levels were both significantly lower in the EMR and PO patients compared with the GA group, reflecting higher TNM stages as well as more like hood to suffer from pyloric obstruction or bleedings and thus with worse nutritional status. Adjuvant chemotherapy was administered to the majority of the patients (570 cases, 63.3%). The percentages of patients receiving adjuvant chemotherapy in the GA, EMR and PO group were 63.2%, 69.3% and 70.0%, respectively, without significant difference. Neo-adjuvant chemotherapy was administered to 4.5%, 6.7% and 2% of the GA, EMR and PO cases, also without significant difference (Table 1).

### Short-term results

The overall morbidity and 30-day mortality for the entire study cohort were 9.6% (86/893) and 0.7% (6/893), respectively. Morbidity occured more commonly in the EMR group with a rate of 25.3% *vs* 8.1% in the GA group (*P* < 0.001) respectively, but was comparable with the PO group (10.0%, *P* = 0.14). And the 30-day mortality for the GA group was 0.4% (3/798), for the EMR group 3.7% (2/75), and for the PO group 5% (1/20) (*P* = 0.004) (Table 1). The 90-day mortality was higher in the EMR (5.3%) than in the GA group (1.6%) (*P* = 0.03), but similar in the PO patients (10.0%) (*P* = 0.45). Peri-operative mortality occurred in 6 patients: 3 subjects in the GA group (0.4%), 2 in the EMR group (2.7%), and 1 in the PO group (5.0%). Significant higher peri-operative mortality was found in the EMR group compared with the GA group (*P* = 0.01). Severe complications, defined as Clavien–Dindo classification ≥ IIIa, occurred in 10 patients in the EMR group (13.3%), which was significantly higher than in the GA group (3.1%, *P* < 0.001) but similar to the PO group (5.0%, *P* = 0.30) (Table 1, Table 2).

**Table 2:** Post-operative complications determined by the Clavien–Dindo classification

	GA group (*n* = 798, 89.4%)	EMR group (*n* = 75, 8.4%)	PO group (*n* = 20, 2.2%)	χ^2^ test	*P*-value
Local complications	44 (5.5%)	11 (14.7%)	0	11.28	0.004
Abdominal infection	20	4	0		
Anastomotic fistula	4	3	0		
Intestinal obstruction	7	1	0		
Abdominal hemorrhage	2	1	0		
Gastrointestinal hemorrhage	5	0	0		
Disruption of wound	2	1	0		
Lymphatic fistula	2	0	0		
Pancreatic fistula	0	1	0		
Duodenal stump fistula	1	0	0		
Anastomotic stricture	1	0	0		
Systemic complications	21 (2.6%)	8 (10.7%)	2 (10.0%)	15.81	<0.001
Pulmonary infection	15	5	1		
Urinary infection	1	0	0		
Pneumothorax	2	0	0		
Renal failure	1	0	0		
Diabetic ketoacidosis	1	0	0		
Cardio- and cerebro-vascular event		3	1		
Total complications	65 (8.1%)	19 (25.3%)	2 (10.0%)	23.28	<0.001
Clavien-Dindo classification				36.05	<0.001
II	40	9	1		
IIIa	7	2	0		
IIIb	11	3	0		
IVa	3	2	0		
IVb	1	1	0		
V	3	2	1		

GA, gastrectomy alone; EMR, extended multiorgan resection; PO, palliative operation.

### Time to recurrence

**Figure 1:**
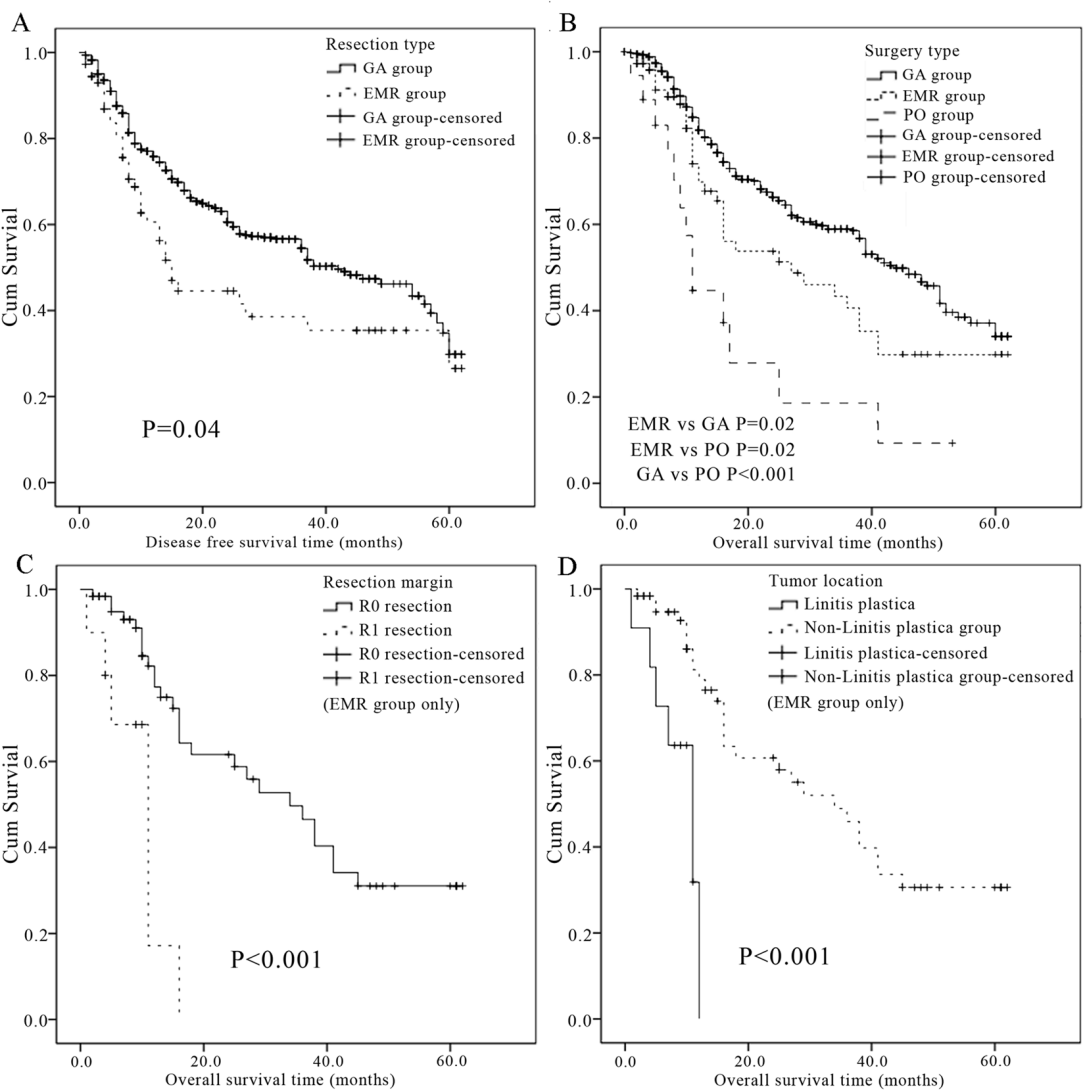
(**A**) Disease free survival cures based on the extent of resection. 798 patients underwent gastrectomy alone (GA group), and 75 patients underwent extended multiorgan resection (EMR group). (Kaplan–Meier procedure, log rank test, *P* = 0.040). (**B**) Overall survival cures based on the extent of resection. 798 patients underwent gastrectomy alone (GA group), 75 patients underwent extended multiorgan resection (EMR group), and 20 patients had palliative operations (11 palliative gastrectomy and 9 gastrojejunostomy, PO group). (Kaplan–Meier method, log rank test. *P* = 0.020 between GA and EMR group; *P* = 0.020 between EMR and PO group; *P* < 0.001 between GA and PO group.). (**C**) Overall survival cures of patients with non-curative resection (R1, *n* = 10) or curative resection (R0, *n* = 65) in the EMR group. (Kaplan–Meier method, log rank test, *P* < 0.001 between the 2 groups). (**D**) Overall survival cures of patients with linitis plastica (*n* = 11) or other type of gastric cancer (*n* = 64) in the EMR group. (Kaplan–Meier method, log rank test, *P* < 0.001 between the 2 groups).

The median follow-up time for patients was 32 months (range, 1–62 months). Recurrence were seen in 36.9% (322/873) of the patients who underwent resection with curative intent (patients in the GA and EMR group). The incidence of recurrence in the EMR group (34/75, 45.3%) was significantly greater than in the GA group (288/798, 36.1%, *P* = 0.040, Fig. 1A). The median time to recurrence was 17 months in the EMR group compared with 41 months in the GA group (*P* = 0.005).

### Survival

The median disease free survival time was significantly longer in the GA compared to the EMR group (*P* = 0.04). The median survival time for the entire cohort of patients was 20 months. The median survival time of the EMR group (27 months) was significantly shorter than the GA group (44 months, *P* = 0.020), but significantly longer than the survival time of the PO group (11 months, *P* = 0.020) (Fig. 1B). The overall 1-year, 3-year and 5-year survival rates were 69.8%, 40.6% and 29.8% respectively in the EMR group, which were statistically worse than in the GA group (84.8%, 58.5%, 34.0%, *P* = 0.020), and as expected, better than the PO group (44.7%, 9.3%, 0, *P* = 0.020).

### Focusing on the EMR group

The clinicopathological characteristics of the 75 patients in the EMR group are summarized in Table 1. The organ that was most frequently resected was the colon (*n* = 17), followed by the liver (*n* = 15) and pancreas/spleen (*n* = 13) (Table 3). More than 1 organ simultaneous resection occurred in 22 cases (29.3%) and the remaining 53 patients underwent only 1 organ resection. pT4b disease (directing invaded to the nearby organs) was confirmed in 53 patients (70.7%) by post-operative histopathological examination, whereas the remaining 22 patients (29.3%) were identified as pT4a (18 cases) or pT3 (4 cases). Although all EMR surgeries were performed with a curative intent, the performance of R0 resection was accomplished in 65 patients (86.7%), while microscopic (R1) residual disease was obtained in the remaining 10 patients (13.3%) (Table 1).

The median survival time was significantly greater in those patients who underwent curative resection (34 months, R0) than in patients with microscopic residual resection (11 months, R1). Therefore, resections significantly influenced survival (*P* < 0.001, Fig. 1C). Patients with linitis plastica had a median survival time of 11 months, which was statistically significantly shorter than the 33 months in patients with non-linitis plastica (*P* < 0.001, Fig. 1D).

The type of organ resected was evaluated for the time to recurrence but no differences were found between pancreatectomy and the resection of other organs. Neither was the number of organs resected a predictor of survival in the entire cohort of 75 patients.

**Table 3:** Frequencies of additional organ resections besides gastrectomy in the EMR group (n = 75).

Organ resected	Frequency
Colon	17 (22.7%)
Liver	15 (20.0%)
Pancreas, spleen	13 (17.3%)
Spleen	11 (14.7%)
Pancreas	10 (13.3%)
Pancreaticoduodenectomy	2 (2.7%)
Pancreas, colon	2 (2.7%)
Pancreas, spleen, liver	2 (2.7%)
Spleen, colon	1 (1.3%)
Liver, colon	1 (1.3%)
Pancreas, spleen, liver, colon	1 (1.3%)

Overwhelmingly after multivariate analysis of the data based on univariate analysis results (Table 4), positive surgical margin (R1 resection, 95% CI, 1.551–10.405, *P* = 0.004) and linitis plastica (95% CI, 1.293–2.502, *P* = 0.019) were shown to be independent predictors for survival after EMR (Table 5).

**Table 4:** Univariate analysis of prognostic factors for overall survival after gastrectomy with curative intent for locally advanced gastric cancer with extended multiorgan resection (EMR group, n = 75).

	Median survival (months)	Log rank test	*P*-value
Age ≥60/<60 years	29/25	1.00	0.32
Gender (male/female)	29/25	0.59	0.44
Complication due to the tumor (yes/no)	25/38	1.18	0.28
Preoperative haemoglobin <35 g/L (yes/no)	18/38	1.55	0.21
Preoperative anemia (haemoglobin <90 g/L) (yes/no)	16/38	2.31	0.13
Gastrectomy type (total/subtotal)	13/41	7.79	0.01
Tumor location (linitis plastica/not)	11/33	16.03	<0.001
Intraoperative blood loss > 400 mL (yes/no)	15/36	3.02	0.08
Operation time > 240 min (yes/no)	16/29	0.03	0.86
Involving pancreas (yes/no)	27/29	0.02	0.90
Non-curative resection/curative resection (R1/R0)	11/34	15.29	<0.001
Number of organs resected (1/ ≥ 2)	29/27	0.18	0.67
Tumor diameter > 7 cm	16/36	1.55	0.21
T stage (T3-T4a/T4b)	38/27	0.40	0.53
N stage (N0/N + )	45/25	3.32	0.07
Metastatic lymph node ratio ≥ 0.5 (yes/no)	13/34	3.81	0.05
Differentiation type (poor/well-moderate)	16/45	3.49	0.06
Postoperative morbidity (yes/no)	13/34	3.90	0.05
Perioperative blood transfusion (yes/no)	25/27	0.004	0.95

**Table 5:** Multivariate analysis of prognostic factors for overall survival after gastrectomy with curative intent for locally advanced gastric cancer with extended multiorgan resection (EMR group, n = 75).

	95% Confidence Interval (CI)	Hazard Ratio	*P*-value
R1 resection	1.551–10.405	4.017	0.004
Linitis plastica	1.293–2.502	1.799	0.019

## Discussion

Gastric cancer usually presents at an advanced stage in the West, China and in undeveloped countries^12,13^. Bleeding and pyloric obstruction were commonly associated with these patients. Unfortunately, of those patients with advanced gastric cancer, especially those who presented with pyloric obstruction, nearly half showed direct invasion of adjacent organs^14,15^. For gastric cancer invading adjacent organs, EMR may be warranted to achieve tumor clearance^16^. On the other hand, with improvement of surgical techniques and perioperative managements, the morbidity decreased. More importantly, the higher morbidity did not translate into higher perioperative mortality^7^. Several studies have confirmed that curative resection was beneficial to patients with advanced gastric cancer and EMR can be performed in carefully selected patients with acceptable morbidity and mortality^3,7,17^. More and more studies have advocated that EMR should be used to treat patients with locally advanced gastric cancer, being the only choice for achieving R0 resections, which is arguably the most significant prognostic factor for the management of gastric cancer^7,18,19^. As far as we are aware, there is a paucity of data that directly compared the safety and efficiency of EMR with gastrectomy alone or palliative surgery for gastric cancer. Moreover, some patients classified as TNM stage IV^3,4,20^, or experiencing distress from additional procedures due to ailments other than tumor invasion, such as simultaneous chlolecystectomy for gallstones^3^ were enrolled into the EMR group, which may affect the credibility of the conclusions. In this large-scale retrospective study, we divided locally advanced gastric cancer patients into EMR, GA and PO groups by surgery approaches for the first time and directly compared the safety and efficiency among the 3 groups.

As reported in previous studies^3,6,7,19^, patients undergoing EMR experienced a higher risk of postoperative complications in the present study, as did the severe complications classified as Clavien ≥ IIIa. Perioperative death was statistically more common in the EMR group (2.7%) compared with the GA group (0.4%, *P* = 0.01). The median survival time of the EMR group in the present study was 27 months, which was similar to previously reported values^3,7,16,19,20^. Though the survival was worse than that in the GA group (44 months), it was significantly better than that in the PO group (11 months). Focusing on the 65 patients undergoing R0 resection in the EMR group, the median survival reached 34 months. Thus, EMR was associated with a satisfactory long-term outcome and acceptable morbidity and mortality for locally advanced gastric cancer in carefully selected patients.

As demonstrated in the present study, a powerful prognostic parameter is R0 resection, which has been widely confirmed by previous studies. The median survival in patients with R0 resection was 34 months, which decreased to 11 months for patients with R1 resection (*P* < 0.001). Although the majority of previous studies advocated carrying out EMR for locally advanced gastric cancer in pursuit of negative margins, a significant proportion of patients had positive microscopic or macroscopic margins (range 11.3–61.6%)^2,6,7,13,19^. The present study confirmed these results with a R1 resection rate of 13.3%. Given the high risk of postoperative morbidity and poor survival, palliative EMR should be avoided as much as possible. To our knowledge, for the first time, linitis plastica has been identified as an independent predictor for survival. Given the diffuse nature and high incidence of peritoneal disease, achieving R0 resection and satisfactory prognosis seems difficult in linitis plastica patients^21,22^. Three patients (27.3%) were found as R1 resection and the median survival for the 11 linitis plastica patients (11 months) undergoing EMR was the same as those undergoing palliative surgery in our study. The extremely poor prognosis of linitis plastica has led others to suggest that it is not a disease requiring surgery with many oncology physicians being against surgical resection for gastric linitis plastica^23^. Although some strictly selected linitis plastica patients who underwent optimal resections^24^ and multimodality therapy^25^ seemed to have an improved long-term survival, the selecting criteria had not been well designated. Thus performing EMR for patients with linitis plastica should be cautiously considered regarding the increased morbidity risk and poor survival. Some studies have demonstrated that tumor size^2^, the depth of invasion^3,19^, lymph node metastasis^3,16,19,20^ and number or type of simultaneous resected organs^7,16,20^ were associated with poor prognosis. However, in the present study, we did not find that any of the above parameters were correlated with survival.

Only 53 patients (70.7%) were confirmed with pT4b disease by postoperative pathological examinations, while 29.3% of the patients had T4a or T3 disease. Our findings are consistent with prior studies that demonstrated that the percentage of patients with confirmed pathological T4 disease ranged from 13.8% to 89% in those undergoing EMR with the goal of R0 resection^3,7,20,26–28^. The precise identification of T4 disease can be difficult by preoperative CT or endoscopic US. In a research project that evaluated the role of CT in the preoperative assessment of T stage, the predictive value in identifying T4 disease was found to be ≤50%^27^. In a recent multi-institutional analysis, the accuracy of endoscopic ultrasound for gastric cancer was found to be only 46.2% for T stage and 66.7% for N stage^29^. It can also be difficult to identify correctly T4 disease by intraoperative assessment. Colen *et al*.^27^ reported that the incidence of pathologically confirmed T4 cancers was only 38.1% (8 in 21) by intraoperative assessment. Given the significantly poorer survival in patients undergoing R1 resection and not sufficiently accurate assessment, it seemed reasonable to perform EMR to achieve R0 resection.

Surprisingly, patients undergoing PO experienced a 30 day mortality rate of 5.0% and a 90 day morbidity rate of 10.0%, which was comparable with those undergoing EMR, though the burden of surgery was smaller in the PO group due to less intraoperative blood loss and shorter operation time and none of them experienced surgery-related complications. A possible explanation might be that the patients in the PO group suffered from pyloric obstruction or bleeding, which may affect the patients’ general condition thereby leading to higher risk of systemic complications. On the other hand, accidental factors may have had an impact on the result in the PO group since they comprised a relatively small number of patients.

The present study had several limitations. First, we tried our best to enroll only patients undergoing resection of the adjacent organs and gastrectomy owing to doubts on the invasion of these organs into the EMR group. We could not precisely exclude some other conditions such as iatrogenic injury, or to facilitate extensive lymphadenectomy as this study was retrospective in nature. Second, patients in the EMR and PO groups were more frequently associated with pyloric obstruction and bleeding, suggesting a systemically advanced tumor and unfortunate clinical outcomes compared to those patients without outlet obstruction even after curative resection^16,30^. Third, patients who underwent either gastrojejunostomy or palliative gastrectomy were enrolled into the PO group and the small sample size in a single institution requires cautious interpretation of the conclusions.

Despite the limitations, the present study provides novel evidence that non-linitis plastica patients undergoing EMR with R0 resection could achieve acceptable postoperative morbidity, mortality and improved survival. Positive residual margin and gastric linitis plastica were associated with poorer survival. These two factors should be borne in mind during the selection process of patients who will most likely benefit from extensive surgery.

## References

[1] Torre LA (2015). Global cancer statistics, 2012. CA Cancer J Clin.

[2] Mita K (2012). Surgical outcomes and survival after extended multiorgan resection for T4 gastric cancer. Am J Surg.

[3] Martin RC, Jaques DP, Brennan MF, Karpeh M (2002). Extended local resection for advanced gastric cancer: increased survival versus increased morbidity. Ann Surg.

[4] Xiao L (2013). Extended multi-organ resection for cT4 gastric carcinoma: A retrospective analysis. Pak J Med Sci.

[5] Okumura Y (2014). Palliative distal gastrectomy offers no survival benefit over gastrojejunostomy for gastric cancer with outlet obstruction: retrospective analysis of an 11-year experience. World J Surg Oncol.

[6] Martin RC, Jaques DP, Brennan MF, Karpeh M (2002). Achieving RO resection for locally advanced gastric cancer: is it worth the risk of multiorgan resection?. J Am Coll Surg.

[7] Tran TB (2015). Multivisceral Resection for Gastric Cancer: Results from the US Gastric Cancer Collaborative. Ann Surg Oncol.

[8] Japanese Gastric Cancer, A. Japanese gastric cancer treatment guidelines 2010 (ver. 3). *Gastric Cancer* **14**, 113–123 (2011). 10.1007/s10120-011-0042-4 21573742

[9] Dindo D, Demartines N, Clavien PA (2004). Classification of surgical complications: a new proposal with evaluation in a cohort of 6336 patients and results of a survey. Ann Surg.

[10] Santiago JM, Sasako M, Osorio J (2011). [TNM-7th edition 2009 (UICC/AJCC) and Japanese Classification 2010 in Gastric Cancer. Towards simplicity and standardisation in the management of gastric cancer]. Cir Esp.

[11] Noh SH (2014). Adjuvant capecitabine plus oxaliplatin for gastric cancer after D2 gastrectomy (CLASSIC): 5-year follow-up of an open-label, randomised phase 3 trial. Lancet Oncol.

[12] Xiao H (2015). Clavien-Dindo classification and risk factors of gastrectomy-related complications: an analysis of 1049 patients. Int J Clin Exp Med.

[13] Carboni F (2005). Extended multiorgan resection for T4 gastric carcinoma: 25-year experience. J Surg Oncol.

[14] Watanabe A (1998). Gastric carcinoma with pyloric stenosis. Surgery.

[15] Lee HJ, Park DJ, Yang HK, Lee KU, Choe KJ (2006). Outcome after emergency surgery in gastric cancer patients with free perforation or severe bleeding. Dig Surg.

[16] Min JS (2012). Prognosis of curatively resected pT4b gastric cancer with respect to invaded organ type. Ann Surg Oncol.

[17] Li MZ (2014). Surgical outcomes and prognostic factors of T4 gastric cancer patients without distant metastasis. PLoS One.

[18] Brar SS (2012). Multivisceral resection for gastric cancer: a systematic review. Gastric Cancer.

[19] Pacelli F (2013). Multivisceral resection for locally advanced gastric cancer: an Italian multicenter observational study. JAMA Surg.

[20] Ozer I (2009). Surgical outcomes and survival after multiorgan resection for locally advanced gastric cancer. Am J Surg.

[21] An JY (2008). Borrmann type IV: an independent prognostic factor for survival in gastric cancer. J Gastrointest Surg.

[22] Kodera Y (2008). The number of metastatic lymph nodes is a significant risk factor for bone metastasis and poor outcome after surgery for linitis plastica-type gastric carcinoma. World J Surg.

[23] Jafferbhoy S, Shiwani H, Rustum Q (2013). Managing Gastric LinitisPlastica: Keep the scalpel sheathed. Sultan Qaboos Univ Med J.

[24] Blackham AU (2016). Is Linitis Plastica a Contraindication for Surgical Resection: A Multi-Institution Study of the U.S. Gastric Cancer Collaborative. Ann Surg Oncol.

[25] Yamashita K (2015). Survival outcome of Borrmann type IV gastric cancer potentially improved by multimodality treatment. Anticancer Res.

[26] Shchepotin IB (1998). Extended surgical resection in T4 gastric cancer. Am J Surg.

[27] Colen KL (2004). Multiorgan resection for gastric cancer: intraoperative and computed tomography assessment of locally advanced disease is inaccurate. J Gastrointest Surg.

[28] Jeong O, Choi WY, Park YK (2009). Appropriate selection of patients for combined organ resection in cases of gastric carcinoma invading adjacent organs. J Surg Oncol.

[29] Spolverato G (2015). Use of endoscopic ultrasound in the preoperative staging of gastric cancer: a multi-institutional study of the US gastric cancer collaborative. J Am Coll Surg.

[30] Chen JH (2007). Outcome of distal gastric cancer with pyloric stenosis after curative resection. Eur J Surg Oncol.

